# 7-Ketocholesterol-Induced Micro-RNA-107-5p Increases Number and Activity of Osteoclasts by Targeting MKP1

**DOI:** 10.3390/ijms23073697

**Published:** 2022-03-28

**Authors:** Guoen Li, Ok-Joo Sul, Rina Yu, Hye-Seon Choi

**Affiliations:** 1 Department of Biological Sciences (BK21 Program), University of Ulsan, Ulsan 44610, Korea; guoen4589@gmail.com (G.L.); suloj@naver.com (O.-J.S.); 2 Department of Food and Nutrition, University of Ulsan, Ulsan 44610, Korea; rinayu@ulsan.ac.kr

**Keywords:** 7-ketocholesterol, micro-RNA-107-5p, osteoclast, mitogen-activated protein kinase phosphatase 1

## Abstract

Osteoclasts (OCs), which are responsible for bone resorption, play a critical role in cholesterol-induced bone loss and recent studies have suggested that various micro-RNAs (miRs) contribute to modulating OCs. We hypothesized that 7-ketocholesterol (7-KC), a metabolite responsible for cholesterol-induced bone loss, induces miR-107-5p, which affects OCs. Overexpression and knock-down of miR-107-5p were performed using miR-107-5p mimic and anti-miR-107-5p, respectively. The effects of miR-107-5p on OCs were analyzed by tartrate-resistant alkaline phosphatase staining, qPCR, and Western blot. MiR-107-5p was upregulated after 7-KC exposure in receptor activator of nuclear factor kappa-Β ligand-stimulated OCs. Furthermore, miR-107-5p upregulation was also observed in tibiae from an atherogenic diet-fed mice compared with mice fed with a normal diet. MiR-107-5p overexpression enhanced the area and number of OCs, whereas inhibiting the endogenous expression of miR-107-5p generated by 7-KC had the opposite effect. Among the possible candidates, mitogen-activated protein kinase phosphatase-1, a stress-responsive dual-specificity phosphatase that inactivates mitogen-activated protein kinase (MKP1), has been proven to be a target gene of miR-107-5p, as demonstrated by the direct interaction between miR-107-5p and the 3′-untranslated region of MKP1. Collectively, our findings demonstrate that 7-KC-induced miR-107-5p promotes differentiation and function of OCs by downregulating MKP1.

## 1. Introduction

Epidemiologic studies have demonstrated that high blood cholesterol contributes to the development of osteoporosis [1,2]. Specifically, hypercholesterolemia has been positively associated with low bone density coupled with an increase of osteoclasts (OCs) in bone tissue of mice [3]. Additionally, statin, a competitive inhibitor of a cholesterol biosynthesis rate-limiting step, increases bone mineral density in humans [4] and protects mice from ovariectomy-induced bone loss [5], suggesting that high cholesterol levels are a risk factor for bone density loss. Our previous studies also demonstrated that an atherogenic diet (AD) induced bone loss in mice by affecting the number and activity of OCs [6]. 

OCs are responsible for bone resorption, originate from hematopoietic progenitors, and are terminally differentiated into multi-nucleated cells. Bone resorption requires mature OCs to tightly adhere to bone and secrete acid and proteases that degrade the bone matrix primarily through the activity of the cross-linking of receptor activator of nuclear factor kappa-Β ligand (RANKL) and receptor activator of nuclear factor kappa-Β (RANK) signaling pathways [7]. Exogenously oxidized cholesterol or oxidized cholesterol metabolites also affect OC activity, as well as OC differentiation [6,8]. OC differentiation and subsequent bone resorption are strongly associated with various signals that affect the tyrosine phosphorylation of a variety of cellular proteins. Protein tyrosine phosphatase (PTP) plays a critical role in modulating these processes, as demonstrated by the high expression of multiple members of the PTP superfamily in OCs [9]. Among them, Src homology region 2 domain containing phosphatase-1 (SHP1), phosphatase and tensin homolog deleted on chromosome 10 (PTEN), and MAP kinase phosphatase 1 (MKP1) act as negative regulators in OCs. SHP1 is a non-receptor-type PTP, which is a product of *Ptpn6*. The bones of mice with naturally occurring inactivating mutations of SHP1, with ~20% the activity of the wild-type (WT) protein, exhibited decreased bone density and an increase in the number and activity of OCs, suggesting that SHP1 participates in bone density loss by affecting the differentiation and activity of OCs [10]. PTEN antagonizes the activity of phosphatidylinositol-3-kinase (PI3K), which inhibits downstream signaling events. Furthermore, PTEN deficiency markedly increased the number of OCs, which coincided with a strong upregulation of NFAT2, suggesting that PTEN acts as an inhibitor of RANKL-induced OC differentiation [11]. MKP1 (gene name, *Dusp1*), a subfamily of dual-specificity phosphatases, is known to downregulate the expression of key genes via the dephosphorylation of p42 MAPK, p38, and JNK [12]. Deletion of MKP1 has been linked to more and larger OCs upon LPS stimulation [13], as well as low bone density in periodontal disease progression [14] and in female mice [15], indicating that MKP1 acts as a negative regulator in OCs. 

Recently, micro-RNAs (miRs) have emerged as a new epigenetic regulator at the post-transcriptional level [16]. These structures constitute a class of 19–25 nucleotide long noncoding RNAs that regulate approximately 60% of human coding genes by binding to the 3′-untranslated regions (UTR) of target genes, resulting in mRNA degradation and transcription inhibition [17]. MiRs have been reported to participate in various kinds of pathophysiological events via regulating cell development, differentiation, proliferation, metabolism, and apoptosis. Several studies have suggested that miRs may also modulate bone mass by controlling OC differentiation and function, thereby modulating bone mass [18,19,20,21,22,23,24,25]. Deficiency in the Dicer protein, which cleaves double-stranded RNA or pre-miR into short double-stranded RNA fragments in OCs, reduces bone resorption [18], suggesting that miR plays a critical role in osteoclastogenesis. MiR-223 targets nuclear factor 1 A-type, resulting in increased OC formation via macrophage colony stimulating factor receptor [19]. Osteoclastogenesis is promoted by miR-183 and miR-21 through repressing heme oxygenase [20] and programmed cell death protein 4 [21], respectively. Conversely, miR-503 and miR-34a inhibit OC differentiation by targeting receptor activator of nuclear factor κΒ [22] and transforming growth factor β-induced factor 2 [23], respectively. Inflammation-induced miR-155 and -29b upregulate autophagy and survival by targeting transforming growth factor β-activated kinase 1-binding protein 2 and Bcl2 modifying factor in OCs, respectively, resulting in inflammatory bone loss [24,25]. 

We hypothesized that 7-ketocholesterol (7-KC), which was upregulated by a high-cholesterol diet, may induce a specific miR that could be responsible for AD-induced bone loss by affecting OCs. Our findings demonstrated that miR-107-5p induced by 7-KC, an oxidized metabolite of cholesterol, affected the number and activity of OCs by decreasing MKP1. 

## 2. Results

### 2.1. Atherogenic Diet Up-Regulates miR-107-5p 

**Figure 1:**
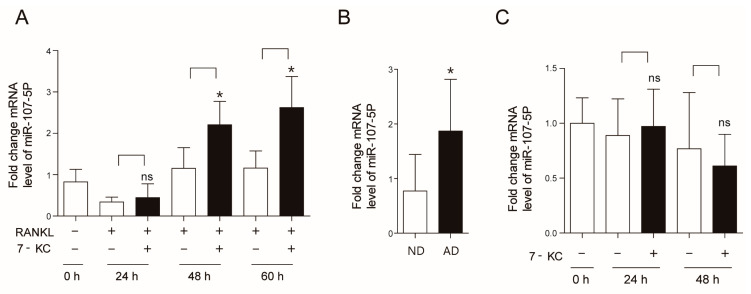
Atherogenic diet (AD) upregulates micro-RNA-107-5p (miR-107-5p). (**A**) Bone marrow-derived macrophages (BMMs) were prepared and incubated with or without 7-ketocholesterol (7-KC, 7 μM) in the presence of macrophage colony stimulating factor (M-CSF, 30 ng/mL) and receptor activator of nuclear factor kappa-Β ligand (RANKL, 40 ng/mL) for the indicated times, and then analyzed by qPCR to quantify the expression of miR-107-5p (n = 3–5). (**B**) Tibiae from mice fed with an AD or normal diet (ND) for 12 weeks were analyzed by qPCR to quantify the expression of miR-107-5p (AD, n = 8; ND, n = 8). (**C**) Primary osteoblasts were treated with 7-KC (7 μM) for the indicated time points and analyzed by qPCR to quantify miR-107-5p expression (n = 4). * *p* < 0.05 compared with each corresponding group; ns, not significant. Similar results were obtained in three independent experiments.

Our previous findings demonstrated that AD induced bone loss, which was accompanied by an increased number and surface of OCs [6]. AD also enhanced the level of 7-KC in tibiae, and 7-KC increased the number and activity of OCs in vitro [8]. We thus hypothesized that 7-KC may regulate various aspects of OCs by modulating multiple miRs. To determine which miR was induced by 7-KC in OCs, miRs associated with cholesterol metabolism were assessed. As shown in Figure 1A, 7-KC increased miR-107-5p expression 1.9-fold after 48 h of exposure and these levels remained high up to 60 h post-exposure, whereas pre-OCs exhibited low levels of miR-107-5p. However, miR-21a-5p, miR-27b-3p, miR-33-5p, miR-125b-5p, and miR-130a-3p had not been modulated by 7-KC among tested miRs (data not shown). Next, to confirm whether these observations could be replicated in vivo, the expression levels of miR-107-5p in the tibiae of AD-fed mice were determined. The level of miR-107-5p in the tibiae of mice fed with an AD for 12 weeks was 2.7-fold higher than in ND-fed mice (Figure 1B). No significant changes in miR-107-5p expression were identified in osteoblasts stimulated by 7-KC up to 48 h (Figure 1C).

### 2.2. miR-107-5p Expression Regulates OC Differentiation

**Figure 2:**
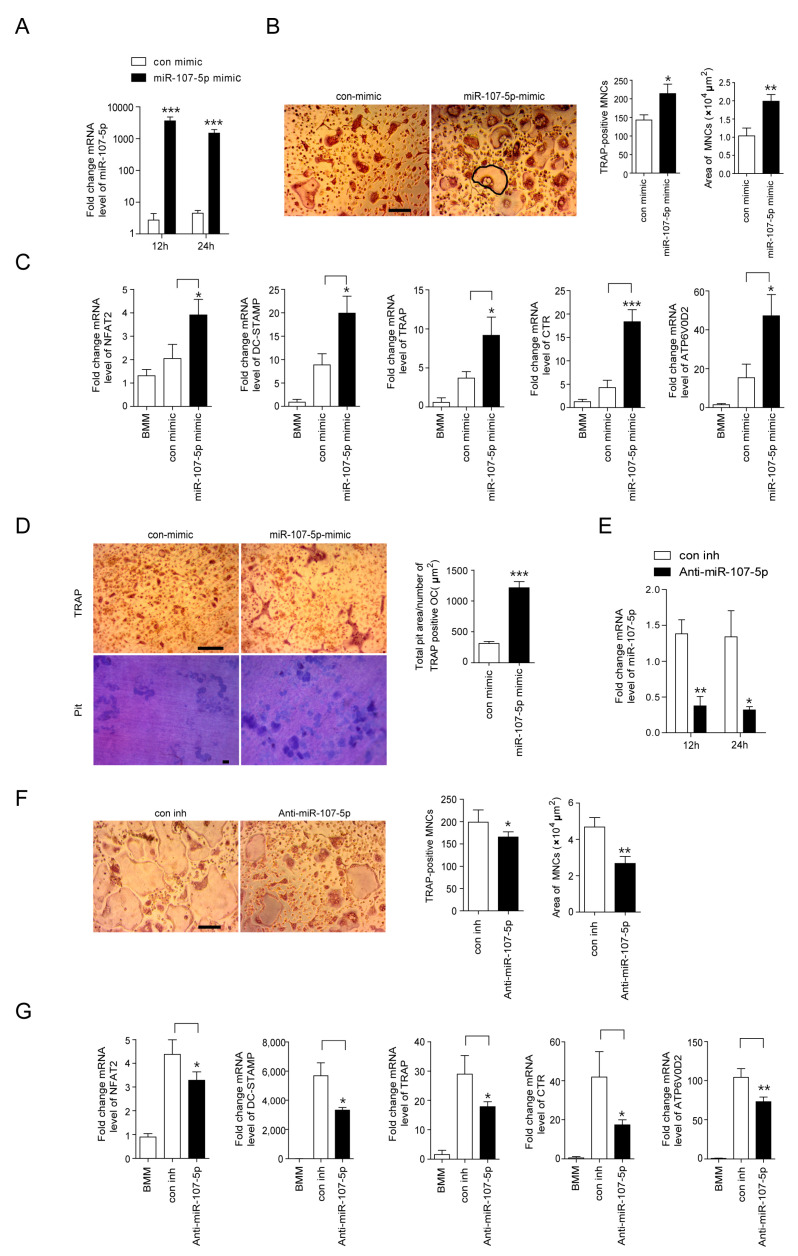
The expression level of miR-107-5p regulates osteoclast (OC) differentiation. BMMs were prepared, incubated with M-CSF (30 ng/mL) and RANKL (40 ng/mL) for 48 h, and transfected with 30 nM of miR-107-5p mimic or con mimic (**A**–**D**). BMMs were prepared, incubated with M-CSF (30 ng/mL), RANKL (40 ng/mL), and 7-KC (7 μM) for 48 h, and transfected with 30 nM of anti-miR-107-5p or con inh (**E**–**G**). After the indicated time points, total RNA was analyzed by qPCR to quantify the expression of miR-107-5p. The expression levels of BMM (**A**,**E**) were set to 1. Cells were incubated for 17 h and fixed to analyze tartrate-resistant acid phosphatase (TRAP)-positive OCs (**B**,**F**) and the expression of TRAP, nuclear factor of activated T cells 1 (NFAT2), calcitonin receptor (CTR), ATP6v0d2, and dendrocyte expressed seven transmembrane protein (DC-STAMP) were quantified by qPCR (**C**,**G**). More than 100 TRAP-positive multinucleated cells (MNCs) in each culture were randomly selected. The area (bold line) of the formed OCs was then measured. Representative photos of OCs. Scale bar; 200 μm (**B**,**F**). Mature OCs were loaded on whole dentine and transfected with miR-107-5p mimic or con mimic for another 5 d in the presence of M-CSF and RANKL. After TRAP staining, the cells were removed, and the slices were stained with toluidine blue. Representative photos of TRAP-positive OCs and resorption pits are shown (scale bar: 50 μm). The total pit area/number of TRAP-positive OCs was calculated (**D**). (A-G; n = 3 each group). * *p* < 0.05; ** *p* < 0.01; *** *p* < 0.001 compared with each corresponding control. Similar results were obtained in three independent experiments.

To evaluate the function of miR-107-5p in OCs, overexpression of miR-107-5p was analyzed in OCs using a miR-107-5p mimicking agent. The level of miR-107-5p in OCs was increased at 12 h after the transfection of the miR-107-5p mimic and remained high up to 24 h compared to the control mimic (con mimic) (Figure 2A). Overexpression of miR-107-5p dramatically increased OC surface area as well as the number of TRAP-positive MNCs (Figure 2B). As illustrated in Figure 2C, miR-107-5p mimic transfection significantly increased the expression of several OC differentiation-associated genes, including nuclear factor of activated T cells 1 (NFAT2), dendrocyte expressed seven transmembrane protein (DC-STAMP), TRAP, calcitonin receptor (CTR), and ATP6v0d2. Given that increasing OC area promotes the activity of OCs, we next evaluated whether miR-107-5p enhanced bone resorption. As expected, overexpression of miR-107-5p significantly enhanced the proportion of the total pit area per TRAP-positive OC loaded in dentine slices (Figure 2D).

Next, the physiological effects of miR-107-5p on the differentiation of 7-KC-induced OCs were evaluated using anti-miR-107-5p to abolish the expression of endogenous miR-107-5p induced upon 7-KC treatment. The transfection of anti-miR-107-5p in 7-KC-treated BMMs significantly decreased the level of miR-107-5p up to 24 h post-transfection (Figure 2E) and lowered the surface area and number of OCs (Figure 2F). Inhibition of miR-107-5p decreased the expression of the OC-specific genes NFAT2, DC-STAMP, TRAP, CTR, and ATP6v0d2 (Figure 2G).

### 2.3. Identification of Target for 7-KC-Induced miR-107-5p in OCs

**Figure 3:**
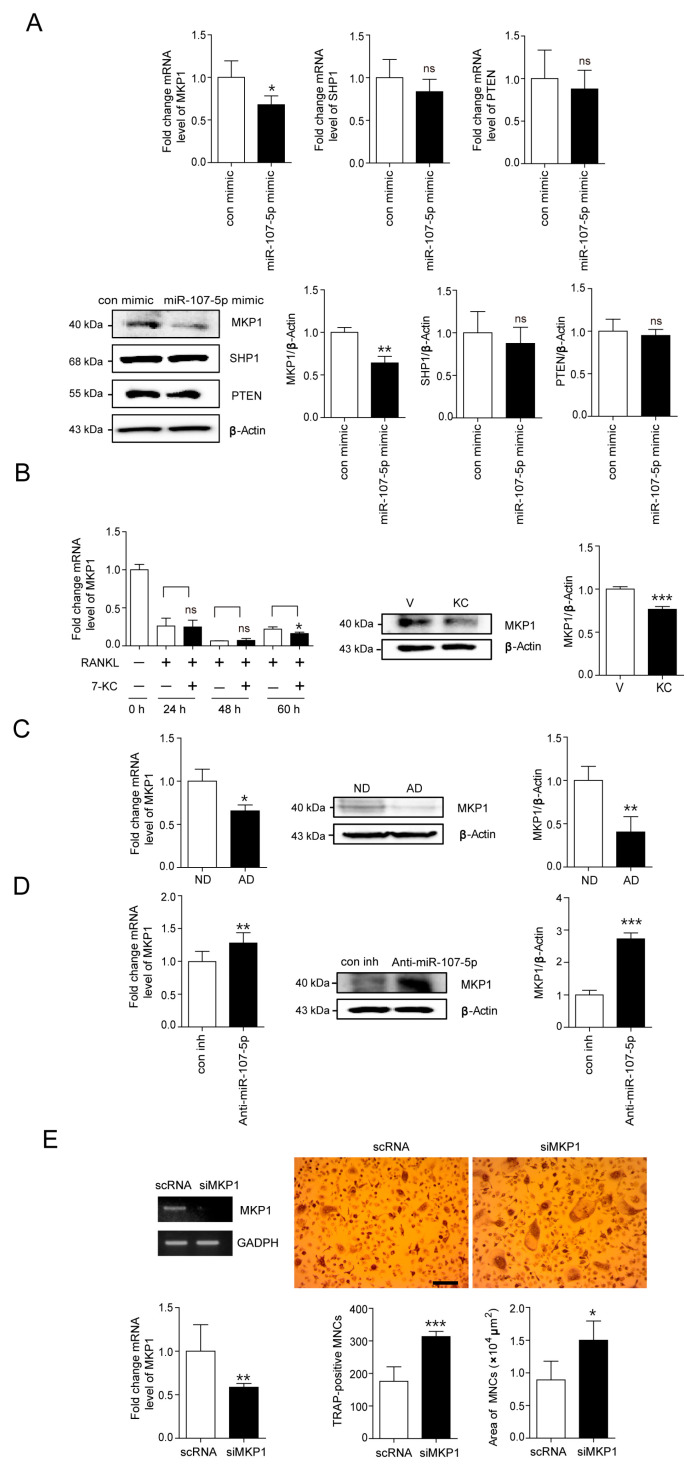
Identification of target for 7-KC-induced miR-107-5p in OCs. (**A**,**D**) BMMs were prepared, incubated with M-CSF (30 ng/mL) and RANKL (40 ng/mL) for 48 h, and transfected with 30 nM of miR-107-5p mimic or con mimic (**A**). BMMs were incubated with M-CSF, RANKL, and 7-KC (7 μM) for 48 h and transfected with 30 nM of anti-miR-107-5p or con inh. (**D**). Cells were incubated for 17 h and total RNA was analyzed by qPCR to quantify the expression of MAP kinase phosphatase 1 (MKP1), Src homology region 2 domain containing phosphatase-1 (SHP1), and phosphatase and tensin homolog deleted on chromosome 10 (PTEN). The expression levels with con mimic treatment (A) or con inh treatment (**D**) were set to 1. Cell lysates were subjected to Western blot analysis with antibodies against MKP1, SHP1, and PTEN. Antibodies against β-actin were used for normalization (n = 4). (**B**) BMMs were incubated with M-CSF, RANKL, and 7-KC. After the indicated time points, total RNA was analyzed by qPCR to quantify the expression of MKP1. After 65 h, cell lysates were subjected to Western blot analysis with anti-MKP1 Ab (n = 3). (**C**) The total RNA and tissue lysates of tibiae from AD- or ND-fed mice were analyzed by qPCR to quantify the expression of MKP1 and were subjected to Western blot analysis with antibodies against MKP1 (AD, n = 8; ND, n = 8). (**E**) BMMs were incubated with M-CSF, RANKL, and 7-KC for 48 h, and transfected with 30 nM of scRNA or si-MKP1 with 7-KC in the presence of M-CSF and RANKL. The cells were incubated for 17 h and analyzed to measure TRAP-positive MNCs. Representative photos of TRAP-positive OCs are shown (scale bar: 200 μm). The silencing of MKP1 by siRNA was confirmed by RT-PCR and qPCR (n = 3). * *p* < 0.05; ** *p* < 0.01; *** *p* < 0.001 compared with each corresponding control; ns, not significant. Similar results were obtained from three independent experiments.

We hypothesized that the molecules associated with OC differentiation signaling pathways could be potential targets of miR-107-5p. Several websites (http://www.targetscan.org [26]; http://pictar.mdc-berlin.de [27], accessed on 16 February 2022) were used to identify the molecules that might be direct targets of miR-107-5p. Several miR-107-5p target candidates were identified, including MKP1, SHP1, and PTEN. Next, qPCR was conducted to evaluate the effects of miR-107-5p on the expression of target candidates. Transfection of miR-107-5p mimic decreased the mRNA level of MKP1 compared to the con mimic, whereas the expression of the other evaluated genes remained largely unaffected (Figure 3A). Overexpression of miR-107-5p also attenuated the protein level of MKP1 (Figure 3A). No significant differences in the protein levels of SHP1 and PTEN were observed compared to the con mimic.

To confirm whether MKP1 could be a target for 7-KC in OCs, the expression levels of MKP1 were evaluated upon 7-KC stimulation. The mRNA level of MKP1 was markedly reduced after 60 h of 7-KC exposure (Figure 3B). The protein level of MKP1 was also attenuated 65 h after 7-KC treatment (Figure 3B). Consistent with our findings in 7-KC-treated OCs, the tibiae of AD-fed mice exhibited a decrease in MKP1 expression at both the mRNA and protein levels compared to ND-fed mice (Figure 3C). Next, we evaluated the effect of blocking endogenous miR-107-5p on the expression of MKP1 in 7-KC-treated OCs. Anti-miR-107-5p transfection significantly enhanced the expression of MKP1 at both the transcript and protein levels in 7-KC-treated OCs compared to con inh (Figure 3D). 

Additionally, to confirm whether 7-KC-enhanced OC differentiation was mediated by attenuating the expression of MKP1, MKP1 silencing was conducted using siRNA. Knock-down of endogenous MKP1 induced by 7-KC increased the OC surface area and the number of TRAP-positive MNCs compared to scRNA transfection (Figure 3E). 

### 2.4. MiR-107-5p Specifically Targets MKP1 by Binding to 3′-UTRs of MKP1 

**Figure 4:**
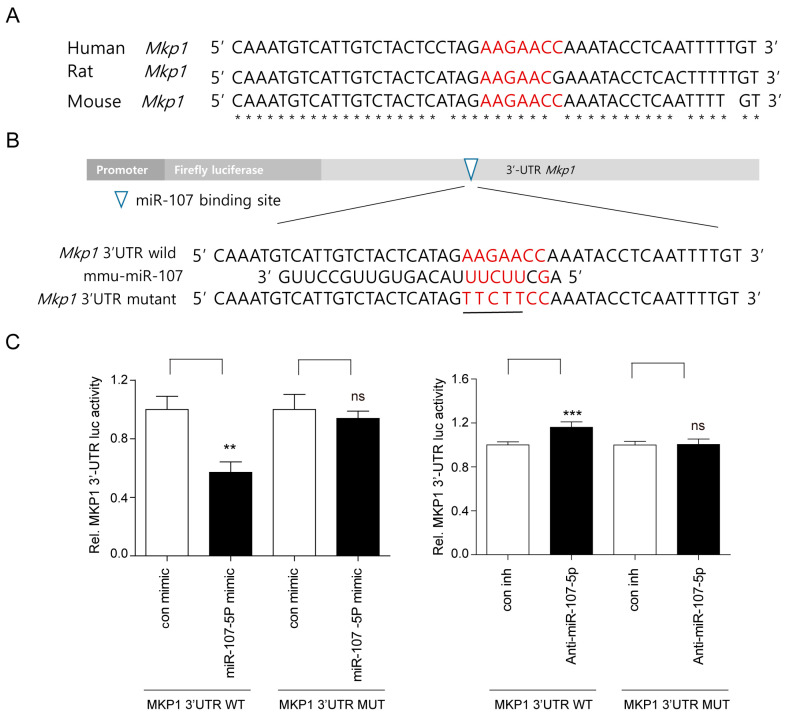
MiR-107-5p specifically targets MKP1 by binding to the 3′-untranslated region (UTR) of MKP1. (**A**) The target site of miR-107-5p in 3′-UTR of MKP1 is conserved in humans and rodents (indicated with asterisks). (**B**) The 3′-UTR mutants of MKP1 are identical to the mouse 3′-UTR wild type (WT) of MKP1 except that it contains five single base substitutions (underlined in the figure) to disrupt pairing with mature miR-miR-107-5p (mmu-miR-107-5p). (**C**) The mouse 3′-UTR WT of MKP1 containing miR-107-5p binding sites or its mutated counterpart were cloned into a luciferase reporter vector and transfected into RAW264.7 cells with miR-107-5p mimic or anti-miR-107-5p with each corresponding control (n = 6). Luciferase assays were performed using a dual-luciferase reporter assay system. ** *p* < 0.01; *** *p* < 0.001 compared to its corresponding control; ns, not significant. Similar results were obtained in three independent experiments.

Bioinformatic analyses demonstrated that the putative miR-107-5p binding site on the 3′-UTR of MKP1 is conserved among several species (Figure 4A). To investigate the direct interaction between miR-107-5p and its potential target gene, MKP1 (*Dusp1)*, the mouse MKP1 WT 3′-UTR containing a miR-107-5p binding site was cloned into a psiCHECK2 vector with a reporter vector containing the mutated 3′-UTR sequences of MKP1 (Figure 4B). After transfection of luciferase reporter vectors containing the WT 3′-UTR of MKP1 and anti-miR-107-5p into RAW264.7 cells, blockade of miR-107-5p enhanced the luciferase activity compared with the inhibition control (con inh), whereas the miR-107-5p mimic diminished it (Figure 4C). However, transfection of the vector with the mutated 3′-UTR sequences of MKP1 did not significantly change luciferase activity (Figure 4C). 

## 3. Discussion 

Our findings demonstrated that 7-KC increases OC differentiation by inducing miR-107-5p, and the tibiae of mice fed with AD exhibited upregulation of miR-107-5p. Increased expression levels of miR-107-5p were detected 48 h after 7-KC exposure in RANKL-stimulated OCs in vitro. Overexpression of miR-107-5p increased the number and surface area of OCs and increased bone resorption, as determined by evaluating the total pit area per TRAP-positive OC, suggesting that miR-107-5p may participate in not only differentiation but also the activity of OCs upon 7-KC stimulation. Conversely, knock-down of endogenous miR-107-5p induced by 7-KC decreased the area and number of OCs. Consistent with our results, upregulation of miR-107 has been found in ovariectomized (OVX) rats, whereas teriparatide treatment had the opposite effect [28], suggesting that miR-107 plays a positive role in bone loss. Furthermore, downregulation of miR-107 has been reported in the livers of leptin-deficient and diet-induced obese mice, resulting in insulin resistance [29]. Our previous findings demonstrated that common factors that induce bone loss in mice can also induce insulin resistance [30,31,32,33], suggesting that miR-107 plays a role in the development of metabolic disease, which could indirectly affect bone density.

According to available websites, the potential targets of miR-107-5p in OCs could be negative signaling molecules such as MKP1, SHP1, and PTEN. We identified MKP1 as a target of miR-107-5p, which in turn is induced by 7-KC stimulation. MiR-107-5p mimic transfection attenuated both the mRNA and protein levels of MKP1 without any significant change in the expression levels of SHP1 and PTEN. Inhibition of endogenous miR-107-5p decreased the expression level of MKP1 in 7-KC-stimulated OCs. Downregulation of MKP1 at both the transcript and protein levels was found in OCs after 7-KC exposure. A similar pattern was observed in the tibiae of mice that were fed with an AD. Luciferase assays using plasmids harboring the 3′-UTR of MKP1 demonstrated the interaction between miR-107-5p and the 3′-UTR of MKP1. Silencing MKP1 in 7-KC-treated OCs resulted in a significant increase in the surface area and number of OCs, indicating that MKP1 plays a critical role in enhancing differentiation and function upon 7-KC stimulation in OCs. Our findings regarding the role of MKP1 in bone resorption are consistent with several previous studies [13,15,34]. MKP1-KO female mice exhibited a decrease in bone mass with fewer OCs and in vitro MKP1 deficiency resulted in increased resorptive activity in OCs [15]. Lack of MKP1 resulted in the formation of more and larger OCs in response to LPS due to an increase in the level of CXCL1 and CXCL2 [13]. In contrast, nanoparticles containing MKP1 agonists significantly reduced bone loss in an LPS-induced periodontitis rat model [34]. 

Taken together, our findings demonstrated that miR-107-5p plays a potential role in mediating the synthesis of 7-KC, an oxidized cholesterol metabolite, by targeting MKP1 to increase the number and activity of OCs. Inhibition of miR-107-5p expression could thus be used as a potential therapy against bone loss caused by a high-cholesterol diet.

## 4. Materials and Methods

### 4.1. Ethics Statement 

All experimental mice were handled according to the guidelines of the Institutional Animal Care and Use Committee (IACUC) of the Immunomodulation Research Center (IRC) at the University of Ulsan. All animal procedures were approved by the IACUC of IRC with the approval ID of #HSC-20-040.

### 4.2. Reagents and Antibodies 

7-KC and a leukocyte acid phosphatase (TRAP) kit were purchased from Sigma Chemical (St. Louis, MO, USA). Recombinant mouse M-CSF and RANKL were acquired from R & D Systems, Inc. (Minneapolis, MN, USA). The mmu-miR-107-5p mimic (miR-107-5p mimic), mmu-miR-107-5p inhibitor (anti-miR-107-5p), control mimic (con mimic), and control inhibitor (con inh), miScript RT kit, and miScript SYBR Green PCR kit were purchased from Qiagen (Hilden, Germany). The Lipofectamine 3000 and Lipofectamine™ RNAiMAX reagents were obtained from Invitrogen (Carlsbad, CA, USA). Primary antibodies against β-actin (A5441) were obtained from Sigma Chemical, whereas MKP1 (sc-373841), PTEN (sc-7974), and SHP1 (sc-287) antibodies were obtained from Santa Cruz Biotechnology (Santa Cru, CA, USA). Small interfering RNA (siRNA) against MKP1 (sc-35938) and scrambled siRNA (scRNA, sc-37007) were obtained from Santa Cruz Biotechnology. M-MLV reverse transcriptase, SYBR Green Real-Time PCR Master Mixes, psiCHECK2, and a dual-luciferase reporter assay system were obtained from Promega (Madison, WI, USA). The QIAzol reagent was purchased from Qiagen (Hilden, Germany).

### 4.3. Animals, Culture of Osteoblast and OC, and OC Formation 

Ten-week-old C57BL/6J male mice purchased from The Jackson Laboratory (Hana, Busan, Korea) were subjected to an AD or chow (normal diet (ND)) for 12 weeks. The AD for mice was purchased from Research Diets, Inc. (New Brunswick, NJ, USA). All mice were housed in a specific pathogen-free animal facility. After the study period, the animals were anesthetized, and tibiae were harvested. Tibiae were dissected free of adherent soft tissue and stored at −80 °C, after which they were homogenized in liquid nitrogen. Total RNA was then extracted using the QIAzol reagent. Primary osteoblasts were prepared from calvarias of C57BL/6J newborn mice, cultured in α-MEM containing 10% FBS for 3 d to confluence as previously described [24], and further incubated with 7-KC. Bone marrow cells were isolated from C57BL/6J male mice as previously described [35]. Femora and tibiae were aseptically removed and dissected free of adherent soft tissue. The marrow cavity was washed with α-MEM from one end of the bone using a sterile 21-gauge needle after dissecting the bone ends, and a single-cell suspension was prepared with a Pasteur pipette. The resulting bone marrow suspension was washed twice and incubated on plates with M-CSF (30 ng/mL) for 16 h. Floating cells were harvested and loaded on a Ficoll-Hypaque gradient and cells at the interface were harvested. Large populations of monocyte/macrophage-like cells adhering to the culture plates were observed after two additional days of cultivation. Floating cells were discarded by washing the dishes with phosphate-buffered saline (PBS), and the adherent bone marrow-derived macrophages (BMMs) were collected and seeded on plates. These cells were analyzed with a FACSCalibur flow cytometer (Becton Dickinson, Franklin Lakes, NJ, USA) and found to be negative for CD3 and CD45R and positive for CD115 [36]. The absence of contaminating stromal cells was confirmed by the lack of growth without the addition of M-CSF. M-CSF (30 ng/mL) and RANKL (40 ng/mL) were added to the cells, and further incubated for the times indicated in the figure legends. After fixation in 10% formalin for 10 min, the cells were stained for tartrate-resistant alkaline phosphatase (TRAP) as described in a previous study [35]. The number of OCs was evaluated by counting TRAP-positive multinucleated cells (MNCs) (with three or more nuclei) per well using an eyepiece graticule at a 100X magnification and the areas of the formed OCs were determined as described [37].

### 4.4. MiR and siRNA Transfection 

BMMs incubated with M-CSF and RANKL in the presence and absence of 7-KC for 2 d were transfected with miR-107-5p mimic and anti-miR-107-5p, respectively, using Lipofectamine 3000 reagent following the manufacturer’s instructions. Afterward, the cells were further incubated for the indicated time with the corresponding control (con mimic and con inh, respectively). BMMs incubated with M-CSF and RANKL in the presence of 7-KC for 2 d were transfected with 30 nM siRNA against MKP1 (siMKP1) or with scRNA using 2 μL of Lipofectamine™ RNAiMAX that was diluted with 50 μL of serum-free media, after which downstream procedures were conducted as described in a previous study [25]. 

### 4.5. RNA Isolation and Quantitative Polymerase Chain Reaction (qPCR) 

RNA extraction and qPCR were conducted as described by Sul et al. [8]. The experiments were conducted using the following primer sequences: 5′-GAC CAC CTT GGC AAT GTC TCT G-3′ and 5′-TGG CTG AGG AAG TCA TCT GAG TTG-3′ (TRAP); 5′-AGT TGC CCT CTT ATG AAG GAG AAG and 5′-GGA GTG TCG TCC CAG CAC AT-3′ (CTR); 5′-AAT AAC ATG CGA GCC ATC ATC-3′ and 5′-TCA CCC TGG TGT TCT TCC TC-3′ (NFAT2); 5′-TTC AGT TGC TAT CCA GGA CTC GGA-3′ and 5′-GCA TGT CAT GTA GGT GAG AAA TGT GCT CA-3′ (ATP6v0d2); 5′-TCC TCC ATG AAC AAA CAG TTC CAA-3′ and 5′-AGA CGT GGT TTA GGA ATG CAG CTC-3′ (DC-STAMP); 5′-ATC AAT GCC AAC TAC GTG AAG AAC-3′ and 5′-GGC TGG CGA TGT AGG TCT TAG A-3′ (SHP1); 5′-AGT GCA GAA TCC GGA TGC A-3′ and 5′-CTG GGC CCC CCT GAT C-3′ (MKP1); 5′-AAT TCC CAG TCA GAG GCG CTA TGT-3′ and 5′-GAT TGC AAG TTC CGC CAC TGA ACA-3′ (PTEN); 5′-ATC AGA GAG TTG ACC GCA GTT G-3′ and 5′-AAT GAA CCG AAG CAC ACC ATA G-3′ (S ribosomal proteins, RPS).

### 4.6. Bone Resorption 

The OCs were further characterized by assessing their ability to form pits on dentine slices as described previously [38]. To this end, mature OCs were generated from BMMs treated with M-CSF (30 ng/mL) and RANKL (40 ng/mL) for 4 d and seeded on dentine slices, transfected with miR-107-5p mimic and its corresponding control (con mimic) using Lipofectamine 3000 reagent, and further incubated for 5 d. The cells were fixed with formalin and stained for TRAP. The cells were then removed via ultrasonication in 1 M NH_4_OH and the dentine slices were stained with 1% (*w*/*v*) toluidine blue in 0.5% sodium borate to visualize the resorption pits. Resorption pit areas and TRAP-positive cells were measured using the ImageJ (version 1.37) software (NIH, Bethesda, MD, USA).

### 4.7. Western Blot Analysis 

After washing with PBS, the cells were treated with lysis buffer (50 mM Tris-HCl, pH 8.0; 150 mM NaCl; 1 mM EDTA; 0.5% Nonidet P-40; 0.01% protease inhibitor mixture). Cell extracts were loaded on SDS-PAGE and transferred onto nitrocellulose membranes. To prevent non-specific binding, the membranes were treated for 1 h with skim milk in Tris-buffered saline containing 0.1% Tween 20, then incubated overnight at 4 °C with antibodies against MKP1, SHP1, PTEN, and β-actin. After washing, the membranes were incubated for 1 h with HRP-conjugated secondary antibodies and developed using chemiluminescence substrates. 

### 4.8. Construction of 3′-UTR reporter of MKP1

Fragments containing the 3′-UTR of MKP1, which included the predicted miR-107-5p binding site, were amplified via PCR. The forward primer was 5′-CTC GAG AGG GCA AGG GGA GGT GTG GAG-3′ and the reverse primer was 5′-GCG GCC GCT CAT CCC AGT AAC AAA ATG TCT-3′. The wild type-psiCHECK2-3′-UTR of MKP1 was obtained by inserting the PCR fragment downstream of the luciferase gene between the Xho1 and Not1 (NEB) sites within the psiCHECK2 luciferase vector, as previously described [24,25]. The mutated 3′-UTR elements of MKP1 were obtained using the PCR-directed mutation method using the appropriate gene-specific forward and reverse primers (5′-TGT CTA CTC ATA GTT CTT CCA AAT ACC TCA-3′ and 5′-TGA GGT ATT TGG AAG AAC TAT GAG TAG ACA-3′, respectively). The mutated PCR fragment was cloned into the same vector and will hereinafter be referred to as Mut-psiCHECK2-3′-UTR of MKP1. All constructs were verified by sequencing.

### 4.9. Luciferase Assays 

RAW264.7 cells were transfected with 30 nM miR-107-5p mimic or anti-miR-107-5p with the corresponding control and 700 ng of WT-psiCHECK2-3′-UTR of MKP1 or Mut-psiCHECK2-3′-UTR of MKP1using the Lipofectamine 3000 reagent. Cells were harvested and lysed after 48 h of transfection. Luciferase assays were conducted using a dual-luciferase reporter assay system. Renilla luciferase activity was used to normalize firefly luciferase activity in each sample.

### 4.10. Statistical Analysis 

All experiments were conducted at least three times and the results are reported as the means ± standard deviation (SD). Pairwise comparisons were conducted using Student’s *t*-test, and multiple comparisons were conducted via one-way ANOVA followed by Bonferroni post hoc tests. A *p*-value < 0.05 was considered statistically significant.

## Data Availability

All original images and data are contained within the article.
